# Nuclear and Mitochondrial DNA Variants in Chiari Malformation Type 1: Insights from an East Coast Malaysian Cohort

**DOI:** 10.5152/eurasianjmed.2026.251209

**Published:** 2026-07-17

**Authors:** Siti Nornazihah Mohd Rosdi, Abdul Aziz Mohamed Yusoff

**Affiliations:** Department of Neurosciences, School of Medical Sciences, Universiti Sains Malaysia, Health Campus, Kubang Kerian, Kelantan, Malaysia

**Keywords:** Chiari malformation type 1, genetic modifiers, mtDNA D-loop mutations, nuclear DNA variants

## Abstract

**Background:**

Chiari malformation type 1 (CM1) is a neurological disorder characterized by cerebellar tonsil herniation. While nuclear DNA has been associated with craniovertebral development, the role of mitochondrial DNA (mtDNA) remains unclear. This study investigated nuclear DNA variants (*PAX1*, *EPAS1*, *DKK1*, *GDF6*) and mtDNA D-loop mutations in CM1 patients from East Coast Malaysia.

**Methods:**

Sixty-eight participants were enrolled, comprising 38 CM1 patients and 30 controls. Genomic DNA from peripheral blood was analyzed by polymerase chain reaction amplification and Sanger sequencing. Associations between genetic variants and clinicopathological parameters (age, sex, tonsillar herniation, syringomyelia) were assessed using chi-square or Fisher’s exact test.

**Results:**

Four nuclear DNA variants were identified, comprising a synonymous PAX1 mutation (c.555G>A, p.K185K) and intronic changes in *EPAS1* (c.1035-7C>G), *DKK1* (c.548-3T>C), and *GDF6* (c.406+112T>C), none of which exhibited significant associations with clinicopathological characteristics. In contrast, mtDNA analysis revealed that 50% of CM1 patients (n = 30) harbored D-loop mutations, predominantly T>C or G>A transitions, including 18 novel variants. Mutation frequency was significantly higher in patients with more severe tonsillar herniation (>10 mm) (OR = 28.570; 95% CI: 3.080-250.000; *P* < .001) and in those with syringomyelia (OR = 7.792; 95% CI: 1.782-34.060; *P* = .007), while no significant associations were observed with age or sex.

**Conclusion:**

Nuclear DNA variants may function as genetic modifiers without significant clinical impact. Conversely, mtDNA D-loop mutations were prevalent and correlated with disease severity, suggesting a contributory role in CM1 pathogenesis and warranting further investigation of mitochondrial genetic factors.

**Main Points**

- Chiari malformation type 1 (CM1) is a structural defect characterized by herniation of the cerebellar tonsils through the foramen magnum. Investigating both nuclear DNA and mitochondrial DNA may improve understanding of the disease mechanisms and their variability.
- No significant association was observed between nuclear DNA variants (*PAX1*,* EPAS1*,* DKK1*,* GDF6*) and clinicopathological parameters, including age, sex, tonsillar herniation length, or presence of syringomyelia, in CM1 patients.
- Mutations in the mitochondrial DNA (mtDNA) D-loop were significantly associated with increased tonsillar herniation and presence of syringomyelia.
- CM1 represents a multifactorial disorder in which nuclear genes may function as modifiers, while mtDNA alterations emerge as pivotal contributors to its pathogenesis, thereby warranting further comprehensive investigation.

## Introduction

Chiari malformations (CMs) were first described in 1891 by Austrian pathologist, Dr. Hans Chiari, who reported a group of structural abnormalities affecting the hindbrain, with severity ranging from mild to severe. These malformations are classified into 4 main types (I-IV), based on the extent of hindbrain tissue that herniates through the foramen magnum.^1^ The mildest form, CM type 1 (CM1), generally develops in individuals who are otherwise born without obvious congenital anomalies.

**Figure 1.**
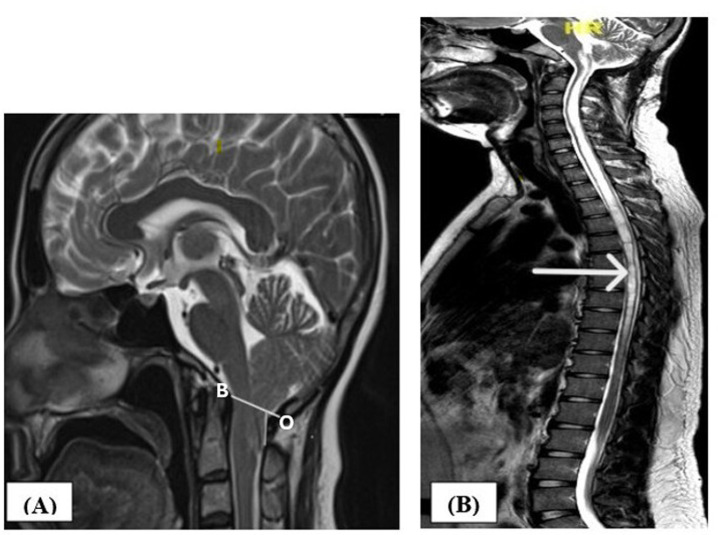
A. Magnetic resonance imaging (MRI) scan of a Chiari malformation type 1 (CM1) patient showing tonsillar herniation extending more than 5 mm below the foramen magnum (the white line denotes the McRae line connecting the basion and opisthion). B. Sagittal T2-weighted MRI image demonstrating cervical-thoracic syringomyelia in a CM1 patient, as indicated by the white arrow.

Chiari malformation type 1 is generally defined as a structural defect in which the cerebellar tonsils extend more than 5mm below the foramen magnum.^1^ Magnetic resonance imaging (MRI) is the primary diagnostic method, with measurements taken on midsagittal images from the tip of the cerebellar tonsils to the McRae line, a line connecting the basion and opisthion (Figure 1).^2^ The estimated prevalence of CM1 in the general population is up to 1%, with a higher occurrence in women than in men and often associated with syringomyelia.^1^ The condition may remain asymptomatic or present with neurological manifestations depending on the degree of tissue compression, nerve involvement, and cerebrospinal fluid (CSF) accumulation in the spinal cord. According to a study by Mueller and Oro,^3^ approximately 98% of CM1 patients experience headaches, often localized to the back of the head, and distinct from migraines or tension headaches. Other symptoms may include vision disturbances, dizziness, neck pain, numbness or tingling in the limbs, difficulty swallowing, and spinal curvature (scoliosis).^4^ Surgical intervention is typically considered when symptoms become persistent or disabling.

Although the precise etiology and genetic mechanisms underlying CM1 remain unclear and are still under investigation, growing evidence suggests that both hereditary susceptibility and environmental factors contribute to its development.^5^ While CM1 is often considered sporadic, emerging research has linked it to genetic influences, with some cases showing familial clustering and nuclear gene mutations, particularly stronger associations involving genes related to collagen production, bone development, chromodomain function, and other developmental processes.^6^

Despite these findings, the role of our second genome, the mitochondrial genome (mtDNA), in CM1 remains unexplored and perhaps deserves greater attention, as mtDNA alterations are well-recognized contributors to numerous disorders. Mitochondria, known as the “powerhouses” of the cell, are essential for adenosine triphosphate (ATP) production, energy regulation, reactive oxygen species (ROS) generation, and the initiation of programmed cell death.^7^ Within the mitochondrial genome lies the displacement loop (D-loop), a 1122 bp non-coding region that is highly susceptible to mutations, exhibits extensive polymorphism, and plays a pivotal role in mtDNA replication and transcription.^7^ This segment has a markedly higher mutation rate than most regions of both the mitochondrial and nuclear genomes, and variations within it have been linked to a range of disorders, including cancers and neurological diseases.^8^ Investigating D-loop mutations in CM1, particularly in familial or atypical cases, could provide valuable insights into its genetic basis and potentially reveal novel variants associated with the condition. Considering the rarity and complexity of CM1, this research focuses on identifying variations in selected nuclear DNA genes (*PAX1*,* EPAS1*, *DKK1*,* GDF6*) and the mtDNA D-loop region among CM1 patients in the East Coast region of Malaysia.

## Material and Methods

### Blood Samples

This case-control study was conducted at the Department of Neurosciences, Universiti Sains Malaysia, between between January 7, 2024 and January 6, 2026, with the research protocol approved by the Research Ethics Committee of Universiti Sains Malaysia (Approval No: USM/JEPeM/KK/23080588; Approval Date: January 7, 2024). The study adhered to the guidelines of the Declaration of Helsinki, and written informed consent was obtained from all the participants prior to enrollment. The study involved 38 peripheral blood samples from patients diagnosed with CM1 and 30 blood samples from healthy volunteers as controls. Inclusion criteria were limited to individuals meeting the diagnostic definition of CM1, characterized by peg-shaped cerebellar tonsils herniating more than 5 mm on MRI. First-degree family members of CM1 patients were also recruited for familial analysis. Patients with tonsillar herniation less than 5 mm were excluded from the analysis. Family members who did not undergo MRI were excluded from analyses involving tonsillar herniation measurements and syringomyelia. However, their blood samples were included in the genetic analysis. Individuals with other forms of CM (types 2, 3, or 4), tonsillar herniation less than 5 mm, significant comorbidities, congenital disorders, or cranial malformations were excluded. For the control group, participants with no clinical or radiological evidence of CM1 and no known family history of the condition were recruited. All samples were stored at −80°C without repeated freeze-thaw cycles to preserve DNA integrity until extraction.

### DNA Extraction

Genomic DNA was isolated from each blood sample using the QIAamp DNA Mini Kit (Qiagen, Germany), according to the manufacturer’s instructions. The yield and purity of the extracted DNA were evaluated using a NanoDrop ND1000 spectrophotometer (Thermo Fisher Scientific, Inc., USA), and its integrity was assessed by 1% agarose gel electrophoresis stained with SYBR^®^ Safe DNA Gel Stain (Thermo Fisher Scientific, Inc., USA).

### Polymerase Chain Reaction Amplification

This study focused on 4 nuclear DNA genes associated with CM1, *PAX1*,* EPAS1*,* DKK1*, *GDF6*, as well as the mtDNA D-loop region. All exons of *PAX1*,* EPAS1*,* DKK1*, and *GDF6* were amplified using primer sets reported in previous studies.^5^ ^,^^9^^-^^11^ The oligonucleotide sequences for the mtDNA D-loop region were obtained from a published reference.^12^

Polymerase chain reaction (PCR) reactions were performed in a 20 µL reaction mixture containing 50-70 ng DNA template, 2U Phusion high-fidelity DNA polymerase, 200 µM of each dNTP, 10 µM of each primer, and 4 µL 5x Phusion HF buffer (Thermo Fisher Scientific, Inc., USA). Thermal cycling conditions consisted of an initial denaturation at 98°C for 30 seconds, followed by 30 cycles of denaturation at 98°C for 10-15 seconds, annealing at 58-67°C for 15-30 seconds, extension at 72°C for 30-50 seconds, and a final extension at 72°C for 5 minutes. Amplified PCR products were separated on 2% agarose gel electrophoresis alongside a Trackit™ 100bp DNA ladder (Thermo Fisher Scientific, Inc., USA) to confirm fragment sizes. PCR products of the expected size were purified using the MinElute® PCR Purification kit (Qiagen, Germany) according to the manufacturer’s protocol and stored at −20°C until sequencing.

### Direct Sanger Sequencing Analysis

Purified PCR products were sequenced in both directions using the same primers employed in the amplification step. Sequencing was performed using a Big Dye Terminator cycle sequencing kit (Applied Biosystems, Foster City, CA, USA) on an ABI Prism 3700 DNA Analyzer automated sequencer, following the protocol outlined by the service provider (Applied Biosystems, Foster City, CA, USA). Electropherograms for the nuclear DNA genes were analyzed and manually aligned using BLAST software available on the NCBI website (http://www.ncbi.nlm.nih.gov/blast). For mtDNA D-loop analysis, sequences were compared to the revised Cambridge Reference Sequence (rCRS) for human mtDNA (NC_012920) using the MITOMAP database (www.mitomap.org).

### Statistical Analysis

All statistical analyses were performed using IBM SPSS Statistics for Windows, version 27 (IBM Corp., Armonk, NY, USA). Descriptive statistics were expressed as mean ± standard deviation (SD) and as percentages for variables following a normal distribution. Associations between genetic variants (nuclear DNA genes and mtDNA D-loop mutations) and clinicopathological parameters were evaluated among CM1 patients only. Categorical variables, including age, sex, length of tonsillar herniation, and presence of syringomyelia, were analyzed using odds ratios (ORs) with 95% CIs. Logistic regression was applied when cell counts were adequate. For analyses involving small sample sizes or 0 cell counts, Fisher’s exact test was used instead of logistic regression, and odds ratios were calculated using the Haldane–Anscombe correction. A *P* value of <.05 was considered statistically significant. The study included 38 CM1 patients and 30 controls, which allowed the assessment of moderate to substantial genetic influences, although smaller effects might not have been detected.

## Results

### Patients’ Characteristics

Demographic data and clinical data for the participants are summarized in Table 1. A total of 68 individuals were recruited, comprising 38 patients diagnosed with CM1 and 30 participants without CM1 who served as controls. Within the CM1 group, there were 14 males (36.8%) and 24 females (63.2%), while the control group included 8 males (26.7%) and 22 females (73.3%). The mean ± SD age of the CM1 group was 34.32 ± 15.97 (ranging from 7 to 64 years), while the control group had a mean ± SD age of 37.60 ± 9.52 (range 18 to 57 years). Most CM1 patients were adults (n = 27, 71%), followed by 9 pediatric patients (23.7%) and 2 individuals over 60 years (5.3%). All participants in the control group were adults (n = 30, 100%). Regarding tonsillar herniation, 27 CM1 patients (57.9%) had herniation >5 mm below the foramen magnum, while 11 patients (42.1%) had herniation >10mm. No herniation was detected in the control group. Syringomyelia was present in 15 CM1 patients (39.5%) and absent in the remaining 23 patients (60.5%), with no cases observed in the control group. Clinical presentations among CM1 patients varied: 6 patients reported a single symptom, 15 experienced multiple symptoms, and 17 were asymptomatic.

### Mutational Analysis of Nuclear DNA Genes (*PAX1*,* EPAS1*,* DKK1*,* and GDF6*) Variants

**Figure 2.**
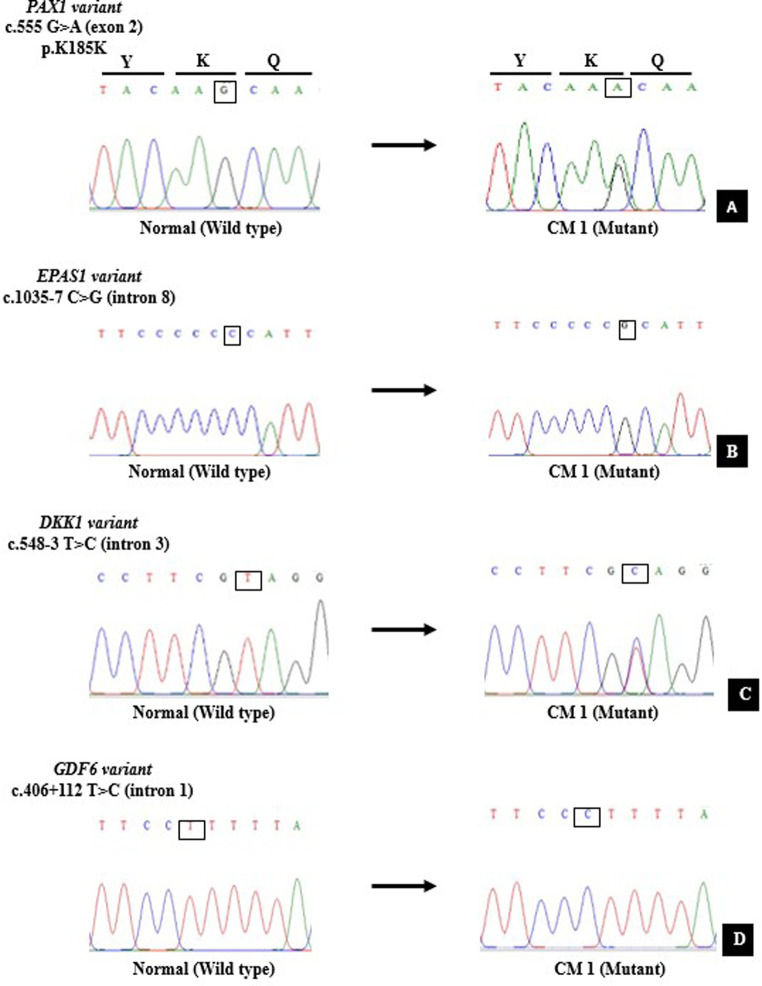
A. Sequencing results of the heterozygous c.555G>A (p.K185K) mutation in the PAX1 gene. B. EPAS1 variant in intron 8, c.1035-7C>G. C. Heterozygous DKK1 variant in intron 3, c.548-3T>C. D. GDF6 variant in intron 1, c.406+112T>C.

A total of 68 participants were screened for potential genetic variants in 4 nuclear DNA genes (*PAX1*,* EPAS1*,* DKK1*,* GDF6*) linked to CM1. Variants were detected in all 4 genes. In the CM1 group, 2 heterozygous variants were identified in *PAX1*,specifically c.555G>A (p.K185K), and were absent in the control group. For *EPAS1*,* DKK1*, and *GDF6*, 1 intronic variant was identified in each gene and was not detected in the control group: c.1035-7C>G, c.548-3T>C, and c.406+112T>C, respectively. Examples of the sequencing chromatograms for these nuclear DNA variants are shown in Figure 2.

### Associations Between Nuclear DNA Variants and Clinicopathological Parameters of Chiari Malformation Type 1

The relationship between these variants and clinicopathological parameters, including age, sex, length of tonsillar herniation, and the presence of syringomyelia, was examined among CM1 patients (Table 2). When mutation frequencies were compared using adult patients as the reference group, no significant differences were observed between pediatric and adult patients across the 4 genes. For *PAX1*, 1 pediatric patient (11.1%) and 1 adult patient (3.7%) carried the mutation, yielding an OR of 3.48 (95% CI: 0.219-55.136; *P* = 0.377). For *EPAS1* and *DKK1*, 1 pediatric patient (11.1%) harbored mutations in each gene, while no mutations were detected among adult patients, resulting in high but imprecise odds estimates (OR = 10.40; 95% CI: 0.360-300.60; *P* = 0.240). Conversely, a single *GDF6* mutation was identified in an adult patient (3.7%) (OR = 0.46; 95% CI: 0.008-27.676; *P* = 0.710). The wide CIs indicate a limited sample size and sparse data. Sex-based analysis, using male patients as the reference group, revealed no statistically significant associations between sex and mutation presence. Mutations in *PAX1*, *EPAS1*, and *DKK1* were observed exclusively among female patients, whereas a *GDF6* mutation was identified in 1 male patient. Female sex was not significantly associated with higher odds of mutation for *PAX1* (n = 2, 8.3%) (OR = 1.091; 95% CI: 0.967-1.231; *P* = 0.522) or for *EPAS1* and *DKK1* (n = 1, 4.2%) (OR = 1.043; 95% CI: 0.960-1.134; *P* = 1.000). Similarly, no significant sex-based association was observed for *GDF6* (n = 1, 7.1%) (OR = 0.929; 95% CI: 0.803-1.074; *P* = 0.368). Patients were further stratified by tonsillar herniation length (>5 mm and >10 mm), with the >5 mm group serving as the reference. For *PAX1*, 1 patient with herniation >5 mm (3.7%) and 1 patient with herniation >10 mm (9.1%) carried mutations. The >10 mm group exhibited lower odds of mutation compared to the >5 mm group, although this association was not statistically significant (OR = 0.385; 95% CI: 0.022-6.757; *P* = 0.501). For *EPAS1*, *DKK1*, and *GDF6*, mutations were detected only in the >10 mm group (n = 1, 4.5% for each gene); however, the odds of mutation did not differ significantly from the reference group (OR = 1.100; 95% CI: 0.913-1.326; *P* = 0.289). The relationship between mutation presence and syringomyelia was also examined, with patients without syringomyelia serving as the reference group. For *PAX1*, mutations were identified in 2 patients with syringomyelia (13.3%), suggesting lower odds of mutation relative to patients without syringomyelia; however, this association was not statistically significant (OR = 0.867; 95% CI: 0.711-1.057; *P* = 0.149). Similarly, mutations in *EPAS1*, *DKK1*, and *GDF6* were detected in only 1 patient with syringomyelia (6.7% for each gene), and no significant associations were identified (OR = 0.933; 95% CI: 0.815-1.069; *P* = 0.395).

### Mutational Analysis of the Mitochondrial DNA D-Loop Region

**Figure 3.**
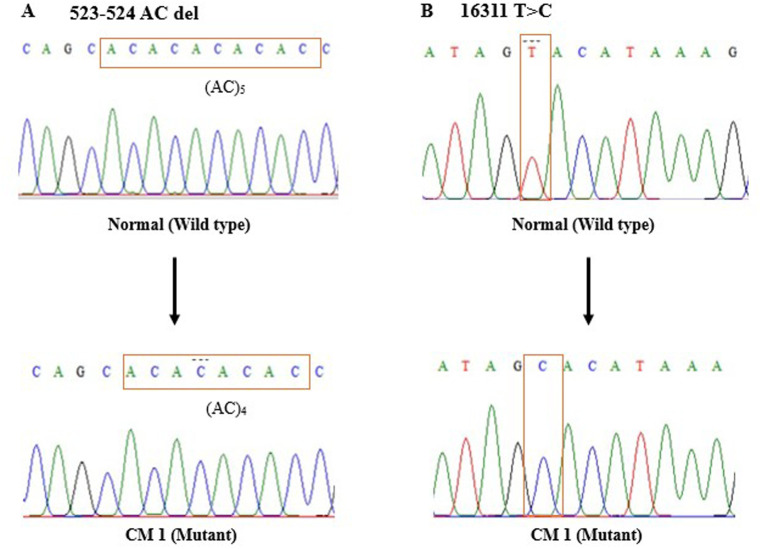
Mitochondrial DNA (mtDNA) D-loop mutations in patients with Chiari malformation type 1 (CM1). A. Deletion of 2 nucleotides (AC) at positions 523-524. B. Homoplasmic mutation involving a T>C transition at nucleotide position 16311.

A complete mtDNA D-loop region was successfully amplified in 68 blood samples. Sequencing results revealed that 19 out of 38 CM1 patients (50%) harbored a total of 30 mutations, spanning 56 nucleotide positions within the D-loop region (Table 3). Most mutations involved T>C or G>A transitions, while 2 positions, 16031 C>G and 16214 C>A, exhibited transversion changes. All detected somatic mutations were homoplasmic. Representative mtDNA chromatograms are presented in Figure 3. Sequence variations detected in both patient and control blood samples were classified as polymorphisms. In this study, 118 polymorphisms were identified at 18 nucleotide positions (Table 4). The comparison of mtDNA D-loop variants between CM1 patients and controls revealed no significant differences in variant frequencies, except for polymorphisms occurring at positions 16519T>C (*P* < 0.001), 489T>C (*P* = 0.009), and the 523-524 AC deletion (*P* = 0.017) (Table 5).

### Associations Between D-loop Mutation Status and Clinicopathological Parameters of Chiari Malformation Type 1

Table 6 summarizes the association between mtDNA D-loop mutation status and clinicopathological parameters, including age, sex, degree of tonsillar herniation, and the presence of syringomyelia among CM1 patients. Using adult patients as the reference group, no statistically significant association was observed between D-loop mutation status and age (OR = 0.286; 95% CI: 0.012-6.914; *P* = 0.286). A sex-based analysis, using male patients as the reference group, revealed that although D-loop mutations were more frequent in female patients (n = 13, 63.2%) than in male patients (n = 4, 28.6%), the odds of mutation presence did not differ significantly between sexes (OR = 0.564; 95% CI: 0.160-1.987; *P* = 0.551). When stratified by tonsillar herniation length, D-loop mutations were detected in 7 of 27 patients (25.9%) with herniation >5 mm and in 10 of 11 patients (90.9%) with herniation >10 mm. Using the >5 mm group as the reference category, patients with tonsillar herniation >10 mm had significantly higher odds of carrying a D-loop mutation (OR = 28.570; 95% CI: 3.080-250.000; *P* < 0.001), indicating a strong association between D-loop mutation presence and greater herniation severity. The association between D-loop mutation status and syringomyelia was also evaluated, using patients without syringomyelia as the reference group. D-loop mutations were significantly more prevalent in patients with syringomyelia (n = 11, 73.3%) than in those without syringomyelia (n = 6, 26.1%), corresponding to higher odds of mutation presence in the syringomyelia group (OR = 7.792; 95% CI: 1.782-34.060; *P* = 0.007).

## Discussion

The pathogenesis of CM1 is acknowledged as complex and multifactorial, encompassing congenital anomalies in cranial and neural development, perturbations in CSF dynamics, and underlying genetic predispositions. Collectively, these factors promote the caudal displacement of the cerebellar tonsils, eliciting clinical manifestations through disrupted CSF circulation and compression of structures.^13^

Milhorat et al^14^ reported that tonsillar herniation in CM1 is associated with hypoplasia of the occipital bone, leading to restricted space within the posterior cranial fossa and subsequent cerebellar crowding. This structural anomaly may be modulated by genes involved in paraxial mesoderm development, particularly *PAX1.* The *PAX1* gene, a highly conserved regulator of embryonic segmentation and vertebral morphogenesis, is essential for normal skeletal formation.^15^ Perturbations in this gene can lead to re-segmentation defects and abnormal vertebral fusion. McGaughran et al^9^ further demonstrated that mutations in *PAX1* are implicated in Klippel–Feil syndrome, a genetic disorder often linked with CM1. In the present study, a heterozygous *PAX1* variant, c.555 G>A (p.K185K), was identified in 2 CM1 patients. According to the Genome Aggregation Database (gnomAD), this variant is classified as benign and has been reported as a single-nucleotide polymorphism (SNP) (rs17861031) in a study in the northern Chinese Han population.^16^ Although this synonymous mutation does not alter the amino acid sequence, it may nonetheless influence gene expression by modulating mRNA stability, splicing, or translational regulation.^17^ Emerging evidence indicates that synonymous mutations can generate cryptic splice sites or disturb gene regulation, as documented in other genetic diseases.^18^ While the functional impact of this variant remains uncertain, its presence suggests a potential contributory role in CM1 pathogenesis.

For *EPAS1*, *DKK1*, and *GDF6*, the identified variants were located within intronic regions. Although introns are typically excised during RNA processing, alterations within adjacent to splice sites may interfere with proper splicing, potentially leading to exon skipping, activation of cryptic splice sites, or intron retention.^19^ The variant identified in *EPAS1* (c.1035-7 C>G) and *DKK1 *(c.548-3 T>C) is situated within splice-region boundaries but not at canonical splice positions, as documented in gnomAD. One human condition linked to alterations in splice sites is Neurofibromatosis type 1 (NF1), an autosomal dominant disorder caused by loss-of-function mutations in the NF1 gene. Approximately 30% of NF1 gene mutations result in splicing changes that affect mRNA processing.^20^ Although these findings do not establish strong statistical significance, the proximity of *EPAS1* and *DKK1 *variants to splice sites suggests a possible contribution to CM1 susceptibility. Accordingly, further functional validation, such as RNA analysis or in-silico splicing prediction, is necessary to clarify the pathogenic relevance of the identified variants. The *GDF6* variant (c.406+112 T>C) has been classified as benign in the ClinVar database and is located within a deep intronic region, more than 100 bp from the nearest canonical splice site. Qian et al^21^ reported that deep intronic splice mutations in inherited retinal disease can alter splicing patterns and impair protein function, accounting for 1.1% of cases in the study. These findings highlight that variants in deep intronic regions should not be disregarded and require further validation to assess their potential contribution to disease susceptibility.

According to the data presented in Table 2, statistical analysis revealed no significant association between nuclear DNA variants (*PAX1*,* EPAS1*,* DKK1*,* GDF6*) and CM1 clinicopathological parameters, including age, sex, length of tonsillar herniation, and the presence of syringomyelia. One possible explanation for the lack of associations is the low frequency of mutations reported in CM1 patients across different cohorts. Speer et al^22^ mentioned that CM1 represents a multifactorial disorder rather than a phenotype driven by a single high-penetrance mutation determining the extent of cerebellar tonsillar herniation. These findings are consistent with those of Markunas et al,^11^ who reported rare functional variants in only 3.8% of CM1 patients. Moreover, the limited identification of pathogenic mutations may be influenced by population-specific genetic architectures. Factors such as founder effects or reduced genetic diversity can contribute to disease clustering in certain ethnic groups. A 2018 study compared CM1 patients across northern and southern regions of the Republic of Tatarstan, Russia, stratified by ethnicity. Among 868 patients, 70% of Tatar ethnicity were disproportionately affected, particularly in the Baltasy (northern) district, where 35% of cases occurred among first-degree relatives. This distribution suggests that genetic or environmental factors may play a contributory role in CM1 pathogenesis.^23^ Similarly, a population-based study conducted in northern New Zealand in 2001 investigated the distribution of CM1 with syringomyelia across different ethnic groups. The cohort consisted of 11.7% Pacific Islanders, 12.5% Maori, and 75.5% Caucasians or other ethnicities. The results demonstrated that syringomyelia associated with CM1 was more frequent among Pacific Islanders (18.4%) and Maori (15.4%), whereas the prevalence was notably lower in Caucasians and other ethnicities (5.4%) per 100 000 population.^24^ Collectively, these findings indicate that the *PAX1*,* EPAS1*,* DKK1*, and *GDF6* variants identified in our study are unlikely to represent direct causative mutations, but may instead function as genetic modifiers that subtly influence cranial development or genetic predisposition to CM1.

A recent case report indicated that a patient with CM1 harbored mitochondrial D-loop variants, suggesting that these variants may contribute to the pathogenesis of the disorder.^25^ Most existing research has focused on nuclear DNA due to its established role in genetic predisposition. In the present study, attention was also directed toward mtDNA, particularly the D-loop region, which is highly prone to mutations. Based on the results of this study, the most frequently detected variants were localized in hypervariable segment 2 (HV2), specifically within the C7TC5 repeat (nucleotides 303-315), a well-recognized hotspot for insertions and deletions.^26^ One CM1 patient exhibited a CCC insertion at nucleotides 303-309, while polymorphisms included C or CC insertions at the same locus. Although the mtDNA D-loop region does not encode proteins, it plays a critical regulatory role in mtDNA replication and transcription, including the control of heavy strand promoter (HSP) regions and replication origins, which are essential for mitochondrial biogenesis.^27^ Variants in this non-coding region may disrupt mtDNA replication fidelity or transcriptional efficiency, thereby contributing to altered oxidative phosphorylation (OXPHOS) and bioenergetic imbalance. Previous studies have demonstrated that alterations in D-loop function are associated with reduced mitochondrial copy number and impaired OXPHOS, suggesting a mechanistic link between D-loop changes and mitochondrial dysfunction in disease states.^28^ Even though mitochondria are central to ATP production through OXPHOS and the regulation of ROS generation, mtDNA exhibits a mutation rate estimated to be tenfold higher than that of nuclear DNA due to its proximity to the electron transport chain, lack of histone protection, and limited DNA repair capacity.^25^ This can lead to a vicious cycle of increased ROS production, further mtDNA damage, and compromised ATP synthesis, as documented in cases of neurodegenerative disorders.^29^ In the context of CM1, decreased ATP supply and elevated oxidative stress may affect tissue development and resilience in the posterior fossa and craniocervical junction, potentially disrupting normal neural and connective tissue homeostasis. This energy deficit could render these structurally critical regions more susceptible to mechanical stress or developmental anomalies underlying tonsillar herniation. Furthermore, abnormal CSF dynamics are well established as a primary pathophysiological factor in CM1. Adequate mitochondrial energy production is essential for maintaining ionic gradients, neuronal signaling, and glial homeostasis, all of which contribute to normal CSF circulation and tissue compliance. Subtle mitochondrial impairment may exacerbate abnormal CSF flow at the craniocervical junction, thereby contributing to disease manifestation or progression.^30^ Collectively, these mechanisms establish a biologically plausible connection between mtDNA D-loop alterations and CM1. In the present cohort, 30 mutations were identified in the D-loop region, of which 12 were previously documented in the MITOMAP database, while 18 represented novel mutations predominantly observed in CM1 cases. In addition, 118 single-nucleotide variations were identified in both patients and controls, all of which were classified as polymorphisms. Prior studies have reported T489C, C16223T, and 523-524 AC deletion as frequent haplotypes among Malaysian populations, with T489C being particularly enriched in Southeast Asian groups.^31^

In the present analysis, the association between mtDNA D-loop variants and clinicopathological parameters in CM1 was investigated. No significant association was observed between D-loop variants and demographic variables such as age or sex. This observation is consistent with a 2014 study, which likewise reported no correlation between mtDNA D-loop variants and age or gender in male patients with obstructive sleep apnea-hypopnea syndrome (OSAHS) (*P *> 0.05).^32^ Furthermore, CM1 patients frequently develop obstructive or central sleep apnea independent of age. However, strong correlations were observed in this study between mtDNA D-loop variants and both the length of tonsillar herniation and the presence of syringomyelia (*P* < 0.05). Godzik et al^33^ reported a significant connection between the extent of tonsillar herniation and the presence of syringomyelia in a cohort of 92 CM1 patients (*P *< 0.001). These findings suggest that the degree of tonsillar descent represents an important indicator of disease severity and further imply that variants may influence the structural and functional manifestations of CM1. A plausible explanation is that alterations within the mtDNA D-loop may hinder mitochondrial performance, resulting in elevated oxidative stress and energy shortages in neural tissues, thereby contributing to the development or progression of syringomyelia. Additionally, Norat et al^29^ suggested that mitochondrial dysfunction exacerbates neuronal injury and compromises brain tissue integrity in a range of neurodegenerative disorders. Collectively, these findings indicate that mitochondrial dysfunction, driven in part by mtDNA alterations, may contribute to CM1 and highlight the need for further investigation into the genetic and molecular mechanisms involved in its pathogenesis.

Limitations, drawbacks, or shortcomings: Attention must be directed to the limitations inherent in this study. First, the sample size was relatively small, which restricted the genetic diversity available for comprehensive mutation analysis. This limitation arose from the difficulty of recruiting patients diagnosed with CM1 and obtaining their blood samples, given the rarity of the condition, which affects approximately 1 in 1000 live births. The restricted cohort size may have influenced the mutation frequencies observed. To address this limitation, future investigations should expand the sampling scope beyond the East Coast of Malaysia to encompass the broader Malaysian population. A larger cohort would enable a more robust evaluation of the relationship between nuclear DNA and mtDNA D-loop alterations in CM1. In addition, the observed association between mtDNA D-loop mutations and clinical manifestations in CM1 highlights the need for a comprehensive analysis of the entire mtDNA genome. Such an approach would enable the detection of mtDNA alterations, including potential protein-altering mutations, thereby offering deeper insights into the molecular mechanisms through which mitochondrial dysfunction contributes to the pathogenesis of CM1. Furthermore, functional validation of identified mutations could be pursued through CRISPR-Cas9 gene editing in animal models, allowing direct observation of the pathogenic impact of specific mutations in vivo. These strategies may uncover novel genetic elements and pathways involved in CM1, ultimately contributing to improved diagnostic approaches and the development of targeted interventions.

In conclusion, this study has advanced the understanding of the genetic contribution of both nuclear and mtDNA in CM1. The variants detected in *PAX1*,* EPAS1*,* DKK1*, and *GDF6 *exhibited no significant correlations with clinicopathological characteristics, suggesting that they may act as genetic modifiers rather than direct pathogenic drivers. In contrast, mutations within the mtDNA D-loop showed a clear association with the extent of tonsillar herniation and the presence of syringomyelia, suggesting that these alterations may impair mitochondrial performance and contribute to disease progression. The discovery of 18 novel D-loop mutations highlights the possible role of mitochondrial genomic instability in the pathogenesis of CM1. Although the relatively small sample size and restricted genetic diversity represent notable limitations, the findings provide a valuable foundation for subsequent research. Expanding the cohort, performing comprehensive sequencing of the entire mitochondrial genome, and conducting functional studies will be essential to elucidate the role of nuclear-mitochondrial interactions in CM1 and to identify potential biomarkers for diagnostic and prognostic applications.

## Figures and Tables

**Table 1.** Demographic and Clinical Characteristics of Study Participants

Parameters	CM1	Control
	(N = 38)	(N = 30)
Sex (n, %)		
Male	14 (36.8)	8 (26.7)
Female	24 (63.2)	22 (73.3)
Age groups (%)		
Paediatric (<18)	9 (23.7)	0 (0.0)
Adult (18-60)	27 (71.0)	30 (100.0)
Over 60	2 (5.3)	0 (0.0)
Mean ± SD	34.32 ± 15.97	37.60 ± 9.52
Length of tonsillar herniation (mm)		
>5	27 (71.1%)	Not assessed
>10	11 (28.9%)	
Presence of syringomyelia, Syr (%)		
Yes	15 (39.5)	Not assessed
No	23 (60.5)	
Clinical manifestations (%)		
Single symptom	6 (15.8)	
Multiple symptoms	15 (39.5)	
No symptoms	17 (44.7)	
Specific symptoms (%)		
Occipital headache	8 (21.1)	
Coughing headache	10 (26.3)	
Bilateral hand numbness	9 (23.7)	
Neck pain	5 (13.2)	
Nystagmus	2 (5.3)	
Bilateral hand numbness and weakness	3 (7.9)	
Oscillopsia	2 (5.3)	
Incontinence	1 (2.6)	
Double vision	1 (2.6)	
Hydrocephalus	3 (7.9)	

CM1, Chiari malformation type 1.

**Table 2.** Association Between Clinicopathological Parameters and Nuclear DNA Variants (*PAX1*, *EPAS1*, *DKK1*, and *GDF6*) in the CM1 Group (N = 38)

Parameters	PAX1			EPAS1			DKK1			GDF6		
	Mutation (%)	No mutation (%)	*P*	Mutation (%)	No mutation (%)	*P*	Mutation (%)	No mutation (%)	*P*	Mutation (%)	No mutation (%)	*P*
Age group												
Paediatric (<18 years)	1 (11.1)	8 (88.9)	.377*	1 (11.1)	8 (88.9)	.240^a^	1 (11.1)	8 (88.9)	.240^a^	0 (0.0)	9 (100.0)	.710*
Adult (18-60 years)	1 (3.7)	26 (96.3)		0 (0.0)	27 (100.0)		0 (0.0)	27 (100.0)		1 (3.7)	26 (96.3)	
Over 60 years	0 (0.0)	2 (100.0)		0 (0.0)	2 (100.0)		0 (0.0)	2 (100.0)		0 (0.0)	2 (100)	
Sex												
Male	0 (0.0)	14 (100.0)	.522^†^	0 (0.0)	14 (100.0)	1.000^†^	0 (0.0)	14 (100.0)	1.000^†^	1 (7.1)	13 (92.9)	.368^†^
Female	2 (8.3)	22 (91.7)		1 (4.2)	23 (95.8)		1(4.2)	23(95.8)		0 (0.0)	24 (100.0)	
Length of tonsillar herniation (mm)												
>5	1 (3.7)	26 (96.3)	.501^†^	0 (0.0)	27 (100.0)	.289^†^	0 (0.0)	27 (100.0)	.289^†^	0 (0.0)	27 (100.0)	
>10	1 (9.1)	10 (90.9)		1 (9.1)	10 (90.9)		1 (9.1)	10 (90.9)		1 (9.1)	10 (90.9)	
Presence of syringomyelia, Syr												0.289†
Yes	2 (13.3)	13 (86.7)	.149^†^	1 (6.7)	14 (93.3)	.395^†^	1 (6.7)	14 (93.3)	.395^†^	1 (6.7)	14 (93.3)

No	0 (0.0)	23 (100.0)		0 (0.0)	23 (100.0)		0 (0.0)	23 (100.0)		0 (0.0)	23 (100.0)	.395^†^

The assumption of normality is fulfilled.

CM1, Chiari malformation type 1.

*Chi-square test.

† Logistic regression test.

a Fisher’s exact test.

a Fisher’s exact test.

**Table 3.** List of mtDNA D-Loop Somatic Mutations Identified in CM1 Patients

Patient code	Nucleotide Position	Somatic Mutation	Homoplasmy/Heteroplasmy	Transition/Transversion
P01	16147	C>T	Homoplasmy	Transition
	145	T>C	Homoplasmy	Transition
	146	C>T	Homoplasmy	Transition
P02	210	A>G	Homoplasmy	Transition
	16031	C>G	Homoplasmy	Transversion
	16145	G>A	Homoplasmy	Transition
	16181	A>G	Homoplasmy	Transition
P03	309	Ins T	Homoplasmy	N/A
	16295	C>T	Homoplasmy	Transition
	16362	T>C	Homoplasmy	Transition
P04	210	A>G	Homoplasmy	Transition
	310	T>C	Homoplasmy	Transition
P05	16362	T>C	Homoplasmy	Transition
P06	16129	G>A	Homoplasmy	Transition
	16311	T>C	Homoplasmy	Transition
	145	T>C	Homoplasmy	Transition
	146	C>T	Homoplasmy	Transition
P07	210	A>G	Homoplasmy	Transition
	309	Ins T	Homoplasmy	N/A
P08	206	Ins A	Homoplasmy	N/A
	125	T>C	Homoplasmy	Transition
	127	T>C	Homoplasmy	Transition
	145	T>C	Homoplasmy	Transition
	146	C>T	Homoplasmy	Transition
P09	185	G>A	Homoplasmy	Transition
	186	A>G	Homoplasmy	Transition
	16399	A>G	Homoplasmy	Transition
P011	145	T>C	Homoplasmy	Transition
	146	C>T	Homoplasmy	Transition
P012	237	A>G	Homoplasmy	Transition
	16311	T>C	Homoplasmy	Transition
	145	T>C	Homoplasmy	Transition
	146	C>T	Homoplasmy	Transition
P013	16209	T>C	Homoplasmy	Transition
	16263	T>C	Homoplasmy	Transition
	16278	C>T	Homoplasmy	Transition
	16319	G>A	Homoplasmy	Transition
P014	16527	C>T	Homoplasmy	Transition
P016	16362	T>C	Homoplasmy	Transition
	16311	T>C	Homoplasmy	Transition
P017	206	Ins A	Homoplasmy	N/A
	125	T>C	Homoplasmy	Transition
	127	T>C	Homoplasmy	Transition
P018	303-309/D310	Inc CCC	Homoplasmy	N/A
P019	204	T>C	Homoplasmy	Transition
	16318	A>G	Homoplasmy	Transition
	16214	C>A	Homoplasmy	Transversion
	16278	C>T	Homoplasmy	Transition
P020	16210	A>G	Homoplasmy	Transition
	16295	C>T	Homoplasmy	Transition
	16362	T>C	Homoplasmy	Transition
	16311	T>C	Homoplasmy	Transition
	145	T>C	Homoplasmy	Transition
	146	C>T	Homoplasmy	Transition
P022	16129	G>A	Homoplasmy	Transition
	16311	T>C	Homoplasmy	Transition

CM1, Chiari malformation type 1; Ins, insertion; mtDNA, mitochondrial DNA; N/A, not applicable.

**Table 4.** mtDNA D-loop Polymorphisms Identified in CM1 Patients and Controls

Nucleotide Position	Base Variation	Cases, n
195	T → C	3
198	T → C	4
303-309/ D310	C Ins	10
303-309/ D310	CC Ins	9
446	C → T	3
489	T → C	12
523-524	AC del	11
16093	T → C	2
16134	T → C	3
16172	T → C	3
16192	C → T	2
16223	C → T	14
16257	C → A	3
16261	C → T	4
16291	C → T	2
16294	C → T	3
16304	T → C	5
16519	T → C	24

del, deletion; Ins, insertion.

**Table 5.** Comparison between mtDNA D-Loop Mutations of CM1 Patients and the Controls

mtDNA D-loop Polymorphism Variant	CM1 (N = 38) (%)	Control (N = 30) (%)	*P*
m.195 T>C	2 (5.3)	1 (3.3)	1.000
m.198 T>C	3 (7.9)	1 (3.3)	.624
m.303-309/ D310 Ins C	8 (21.1)	2 (6.7)	.167
m.303-309/ D310 Ins CC	5 (13.2)	4 (13.3)	1.000
m.446 C>T	2 (5.3)	1 (3.3)	1.000
m.489 T>C	11 (28.9)	1 (3.3)	.009
m.523-524 del AC	10 (26.3)	1 (3.3)	.017
m.16093 T>C	1 (2.6)	1 (3.3)	1.000
m.16134 T>C	1 (2.6)	2 (6.7)	.579
m.16172 T>C	2 (5.3)	1 (3.3)	1.000
m.16192 C>T	1 (2.6)	1 (3.3)	1.000
m.16223 C>T	9 (23.7)	5 (16.7)	.556
m.16257 C>A	1 (2.6)	2 (6.7)	.579
m.16261 C>T	1 (2.6)	3 (10.0)	.314
m.16291 C>T	1 (2.6)	1 (3.3)	1.000
m.16294 C>T	1 (2.6)	2 (6.7)	.579
m.16304 T>C	4 (10.5)	1 (3.3)	.374
m.16519 T>C	20 (52.6)	4 (13.3)	<.001

CM1, Chiari malformation type 1; del, deletion; Ins, insertion; mtDNA, mitochondrial DNA.

**Table 6.** Association Between Clinicopathological Parameters and mtDNA D-Loop Mutation Status in CM1 Patients

Parameter	D-loop Status, N (%)			
	Total Patients, N (%)	Mutation (%)	No Mutation (%)	*P*
No. of patients	38 (100.0)	19 (50.0)	19 (50.0)	
Age group				
Pediatric (<18 years)	9 (23.7)	7 (77.8)	2 (22.2)	.077*
Adult (18-60 years)	27 (71.0)	9 (33.3)	18 (66.7)	
Over 60 years	2 (5.3)	1 (50.0)	1 (50.0)	
Sex				
Male	14 (36.8)	4 (28.6)	10 (71.4)	.181^†^
Female	24 (63.2)	13 (54.2)	11 (45.8)	
Length of tonsillar herniation, (mm)				
>5	27 (71.1)	7 (25.9)	20 (74.1)	<.001^†^
>10	11 (28.9)	10 (90.9)	1 (9.1)	
Presence of syringomyelia, Syr				
Yes	15 (39.5)	11 (73.3)	4 (26.7)	.007^†^
No	23 (60.5)	6 (26.1)	17 (73.9)	

The assumption of normality is fulfilled.

*Chi-square test.

^†^Logistic regression test.

CM1, Chiari malformation type; mtDNA, mitochondrial DNA.

## Data Availability

The data that support the findings of this study are available on request from the corresponding author.
